# Supplementary material for: Complexome profiling of the Chlamydomonas psb28 mutant reveals TEF5 as an early PSII assembly factor
Source: Plant Cell. 2025 Mar 17;37(6):koaf055. doi: 10.1093/plcell/koaf055 (PMC12164590; doi:10.1093/plcell/koaf055)
Supplement: koaf055_Supplementary_Data [file koaf055_supplementary_data.zip › psb28_interactive.zip!/plots/intensity/qualityPlots/peptides/KAR1_Cre03.g172000.t1.2.html]

### Cre03.g172000.t1.2

Trivial name: KAR1  
SeqCoverage [median]: 6.20  
Mass: 33.12 kDa  
  
 

| Sequence | MissedCleavage | Mass [Da] | wtIntensity | muIntensity | Score |
| --- | --- | --- | --- | --- | --- |
| AIALALGK | 0 | 755.49 | 312.04 | 0.00 | 66.99 |
| VDVLVNNAGITR | 0 | 1269.70 | 0.00 | 761.71 | 94.47 |
|  |  |  | 312.0 | 761.7 |  |
